# Identification and Functional Analysis of the *psaD* Promoter of *Chlorella vulgaris* Using Heterologous Model Strains

**DOI:** 10.3390/ijms19071969

**Published:** 2018-07-06

**Authors:** Jongrae Kim, Linpo Liu, Zanmin Hu, EonSeon Jin

**Affiliations:** 1 Department of Life Science, College of Natural Sciences, Hanyang University, Seoul 04763, Korea; kjr1210@hanmail.net; 2 Institute of Genetics and Developmental Biology, Chinese Academy of Sciences, Beijing 100101, China; lip668@126.com (L.L.); zmhu@genetics.ac.cn (Z.H.)

**Keywords:** *Chlorella vulgaris*, native promoter, photosystem I protein D (*psaD*), heterologous expression

## Abstract

*Chlorella* has great potential as a bio-factory for production of value-added compounds. To produce the desired chemicals more efficiently in *Chlorella*, genetic tools for modification of *Chlorella* need to be developed, especially an endogenous promoter. In this study, the promoter of photosystem I protein D (*psaD*) from *Chlorella vulgaris* UTEX395 was identified. Computational analysis revealed the presence of several putative cis-acting elements, including a potential core element, and light-responsive or stress-responsive elements. Gene expression analysis in heterologous expression system in *Chlamydomonas* *reinhardtii* and *Nicotiana* *benthamiana* showed that *CvpsaD* promoter can be used to drive the expression of genes. Functional analysis of this promoter suggested that the initiator element (Inr) is important for its function (i.e., TATA-less promoter) and that an additional factor (e.g., downstream of the transcriptional start site) might be needed for light response. We have shown that the *CvpsaD* promoter is functional, but not sufficiently strong, both in microalgae and higher plant.

## 1. Introduction

To date, microalgae are being commercially used to produce food and animal feed additives, as an ingredient in cosmetics, or as value-added compounds, such as pigments, therapeutic proteins, and fatty acids [1,2,3]. Owing to the recent developments in microalgae transformation techniques, microalgae now have much broader industrial applications; use of transgenic microalgae for production of medicines and biofuels has already demonstrated this [4]. However, the production of the biochemical is still insufficient. Therefore, to improve the industrial potential of microalgae, genetic modifications for enhancement of their physiological properties and optimization of production systems of commercial strains are being conducted by many research groups [5]. The well-known unicellular freshwater green microalga, *Chlorella vulgaris*, is widely cultured and commercialized because of its ability to grow rapidly and economically in both autotrophic and heterotrophic media [6,7]. Although a method for developing transgenic *C. vulgaris* has already been reported [8], the methods cannot be applied to all fields because of its low efficiency or non-reproducible results. Therefore, to use this promising industrial species more effectively, there is a need to develop reliable and convenient tools for genetic modification [9].

Several methods, such as protoplast transformation [10,11], *Agrobacterium*-mediated transformation [12,13], electroporation [14,15], and particle bombardment [14,16] have been developed to transform *Chlorella*. However, some drawbacks are still associated with these methods as inefficient or unstable transformants are produced. The main reasons for the formation of such transformants are as follows: (I) the lack of a high efficiency transfer technique for introduction of a foreign gene into the genome [5], (II) the lack of an appropriate expression system for foreign genes [17,18,19,20], (III) the difference in codon usage between the expression host and the introduced foreign gene [21,22,23].

*Chlamydomonas reinhardtii*, a green microalga, has long been used as a model strain for molecular biology and genetic experiments using its own native promoters. Several native promoters have been identified that are known to regulate well-characterized and highly expressed genes, such as heat shock protein 70A (*HSP70A*), Rubisco small subunit (*RBCS2*), or photosystem I protein D (*psaD*) [24,25,26]. Moreover, in an attempt to increase the expression rate, a chimeric promoter was developed that contains the *HSP70A* promoter region fused upstream of the *RBCS2* promoter (AR promoter), thereby, leading to increased transcription [25,27]. Recently, some research groups suggested the use of synthetic algal promoters (saps) that are designed based on the characteristics of strong promoter motifs [28,29]. While the use of endogenous promoters is almost optimized in the model algal strain, only heterologous promoters from the plant system, such as the 35S promoter, ubiquitin promoter, and NOS promoter have been used for *Chlorella* [10,11,12,15,16]. Therefore, research on promoters derived from *Chlorella* is still in its early stage, and further advancements are necessary.

In general, gene expression is positively correlated with promoter strength. Therefore, the promoter of a gene encoding a highly expressed protein in vivo is considered to be a strong promoter. *psaD* gene encodes an abundant protein of the photosystem I reaction center subunit II in photosynthetic organisms [30,31,32]. *psaD* gene is a nuclear gene although *psaD* protein is located on the stromal side in the chloroplast. The *psaD* promoter has been used to drive efficient gene expression in *Chlamydomonas* [26,32]. Since this gene is commonly found in photosynthetic organisms, it is also found in *Chlorella* species.

In this study, we found the photosystem I protein D (*psaD*) gene from the whole genome sequence (BioProject: PRJNA278897) of *Chlorella vulgaris* UTEX395 and predicted its promoter sequence in the 5′ upstream region of *psaD*. The function of this *CvpsaD* promoter was confirmed through the expression of *aphVIII* or luciferase in *Chlamydomonas reinhardtii* (microalgae) and also the expression of green fluorescent protein (GFP)-fused β-glucuronidase (GUS) in *Nicotiana benthamiana* (higher plant). This is the first report about the use of an endogenous promoter of *Chlorella vulgaris* for transgene expression in other organisms.

## 2. Results and Discussion

### 2.1. Isolation and Computational Analysis of CvpsaD Gene and the 5′ Upstream Region

**Figure 1:**
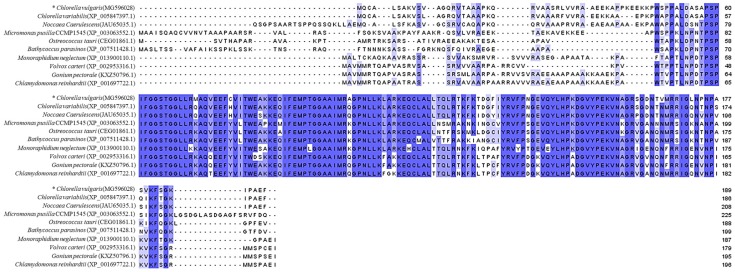
Alignment of amino acid sequence of photosystem I protein D (*psaD*) using the protein basic local alignment search tool (BLASTP) program against the National Center for Biotechnology Information (NCBI) database (https://blast.ncbi.nlm.nih.gov/Blast.cgi). The asterisk indicates the *psaD* sequence of *C. vulgaris* UTEX395 used for this study.

*Chlorella vulgaris* UTEX395 *pasD* (MG596028) gene was predicted by the protein basic local alignment search tool (BLASTP) program using the amino acid sequence of *Chlamydomonas reinhardtii psaD* as the query, and computational analysis was performed using the NEW GENESCAN Web server. Putative *psaD* sequence of *C. vulgaris* was subjected to BLASTP analysis for a sequence homology search against the National Center for Biotechnology Information (NCBI) database. The search results indicated that the conserved domain of *CvpsaD* has a very high similarity with those of other species. Thus, BLASTP analysis and alignment of the conserved domain (*psaD* superfamily, PLN00041) of *psaD* family suggested that the predicted sequence is indeed *psaD* (Figure 1).

**Figure 2:**
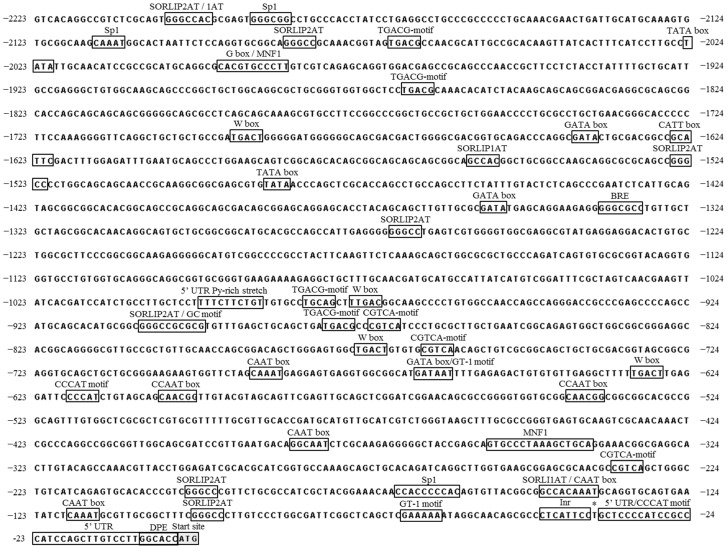
Nucleotide sequence of the *CvpsaD* promoter and its predicted motifs. The start codon (ATG) is highlighted in a gray box and the putative transcriptional start site (TSS) is indicated by an asterisk. The putative cis-acting elements are boxed and labeled.

To determine the putative cis-acting elements, the 2.2 kb putative promoter sequence upstream of the *CvpsaD* protein-coding sequence CDS was analyzed using PLACE and PlantCARE databases. Additionally, the Promoter 2.0 Prediction Server was used to predict the transcription start site (TSS), and 5′-rapid amplification of complementary DNA ends (5′-RACE) was performed to determine the 5′ untranslated region (5′-UTR) sequence. The TSS site was found to be at −36 bp by 5′-RACE (Figure 2), whereas the predicted TSS site by Promoter 2.0 Prediction Server was at −1200 bp. The difference in position between the actual TSS and the predicted TSS site was remarkable. Thus, TSS analysis through computational techniques was not effective in the *Chlorella* promoter study. The TATA-box, the core element, which is typically located 30~40 bp upstream of the TSS site in eukaryotes, was identified at two positions by PlantCARE, but they were quite far away (−1480 bp and −2020 bp) from the TSS. Therefore, TATA-box might not be the core element in the *CvpsaD* promoter. In plants, TATA-less promoters are commonly found in the photosynthetic genes, such as photosystem I subunits [33]. The initiator element (Inr) consensus sequence, YYANWYY, is one of the alternative core elements found in TATA-less promoters [34]. The sequence-based structural features of *CvpsaD* promoter are similar to those of *psaDb* promoter which is a TATA-/Inr+ promoter in *Nicotiana sylvestris* [33]. Their Inr has the same consensus sequence as CTCAYTYY. Therefore, instead of the TATA-box, Inr, located at −38 bp, could play an important role in in mediating the functions of the *CvpsaD* promoter. Moreover, it is expected that a downstream promoter element (DPE, −6 bp) will act as a core element with Inr in the *CvpsaD* promoter [33,35]. BRE, another core element, was found at −1330 bp. This element is located immediately upstream of the TATA-box; thus, it is a non-critical factor in TATA-less promoters, like the *psaDb* and *CvpsaD* promoters. The distal sequence may contain additional regulatory elements, often with a weaker influence than the core element. The CAAT box, which is a common cis-acting element in promoters, was found at −80 bp, −100 bp, −340 bp, and −650 bp from TSS. This motif is normally located 60–100 bp upstream of the TSS and about 150 bp upstream of the TATA box. The 5′ UTR Py-rich stretch motif is also located in the −950 bp upstream region. This 5′ UTR motif can provide high transcription levels without the need for other upstream cis-acting elements [36,37]. Two CCCAT motifs [28], which are putative core elements isolated from synthetic promoters, were observed at −600 bp and +13 bp from TSS. Although these three elements are not key elements, they are located close to the TSS and will have a greater effect on the *CvpsaD* promoter than the TATA-box. Additionally, as shown in Figure 2 and Table 1, the *CvpsaD* promoter contains several light-responsive elements, such as CATT box, G-box [38], GATA box [39], MNF1 motif [24], SORLIP1AT, SORLIP2AT [29,40,41], and the Sp1 motif [42], which are found in higher plants. These light-responsive elements are widely distributed throughout the promoter of *CvpsaD.* Also, there is a report that the *Cppsy* promoter of *Chlorella protothecoide* which has MNF1, Sp1 motif regulated by light [43]. Thus, the *CvpsaD* promoter may respond to light. Simultaneously, other elements associated with stress-responses, like the GT-1 motif [44], W box [45], CCAAT box [46] CGTCA-motif, and TGACG-motif [47], which are found in plants, were also observed in the promoter of *CvpsaD*. These elements are known to be related by methyl jasmonate (MeJA), but the direct association of the *psaD* gene by MeJA has not been reported.

### 2.2. Construction of the CvpsaD Promoter Cassettes and Validation of Its Function in C. reinhardtii

**Figure 3:**
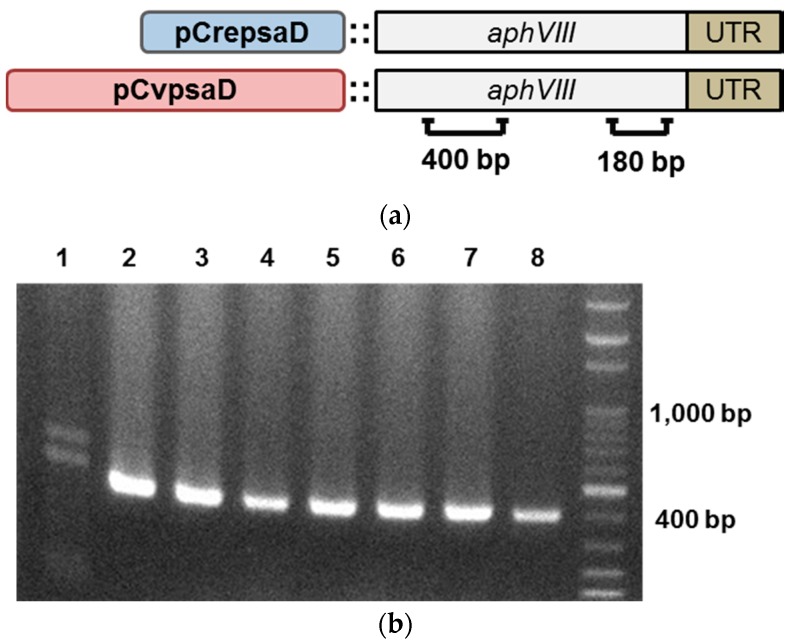

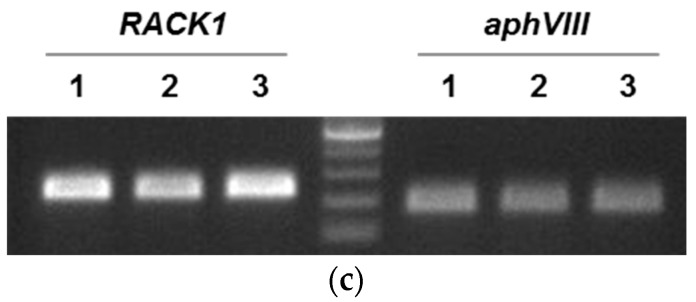
Validation of *CvpsaD* promoter functionality by expression of *aphVIII*. (**a**) Vector map of promoter cassette conjugated with resistance gene for verification of functionality. The *CrepsaD* promoter cassette serves as a positive control in *Chlamydomonas*. (**b**) The presence of *aphVIII* was confirmed in putative transformants by polymerase chain reaction (PCR) analysis with 400 bp primer sets. Lane 1: wild type, Lane 2–4: *pCrepsaD*::*aphVIII*, Lane 5–7: *pCvpsaD*::*aphVIII*, Lane 8: plasmid positive, (**c**) RNA expression was detected by RT-PCR using 180 bp primer sets for *aphVIII*. The *RACK1* gene served as the internal control. Lane 1: *pCrepsaD*::*aphVIII*, Lane 2–3: *pCvpsaD*::*aphVIII*.

The gene encoding aminoglycoside 3′-phosphotransferase (*aphVIII*) was chosen as the selective marker gene as it provides stable resistance against paromomycin, a commonly used selection antibiotic for microalgae. For the construction of promoter cassette containing the desired promoter and selective marker, first the *aphVIII*-3′ UTR fragment was cloned into pChlamy3 plus vector and digested with restriction enzymes (*Spe*I and *Kpn*I), and then the promoter sequence was cloned upstream of *aphVIII*-3′ UTR (Figure 3a). To validate the function of *CvpsaD* promoter, the promoter cassette with *aphVIII* was transformed into *Chlamydomonas*. The *CrepsaD* promoter cassette was used as a positive control. The transformants with paromomycin resistance were selected on agar plates and three different transformants of each promoter cassette were sub-cultured in liquid medium for polymerase chain reaction (PCR) analysis. To confirm that the promoter cassette DNA was introduced into the cells, PCR was performed with specific primers (400 bp product, Figure 3a,b). The expression of *aphVIII* RNA in transgenic *Chlamydomonas* was determined by reverse transcription PCR with specific primers (180 bp product, Figure 3a,c). Unless the antibiotic resistance genes introduced into microalgae are expressed sufficiently to resist against antibiotics, transformants will be not produced [48]. Thus, from this result, we found that *aphVIII* was highly expressed by the *CvpsaD* promoter, at levels sufficient enough to confer antibiotic resistance in *Chlamydomonas*.

### 2.3. Validation of the CvpsaD Promoter Efficiency in C. reinhardtii

**Figure 4:**
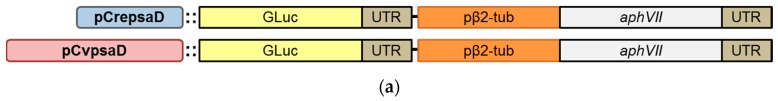

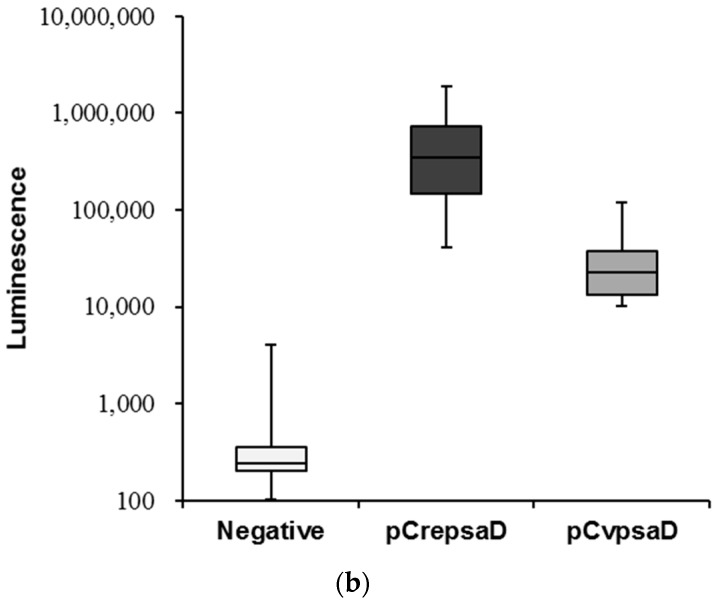
Determination of the strength of *CvpsaD* promoter. (**a**) Schematic representation of the promoter cassettes conjugated with *Gaussia* luciferase (GLuc) gene for strength analysis (**b**) Analysis of luciferase activity by the two promoters.

To determine the strength of *CvpsaD* promoter, a new vector cassettes for *Gaussia* luciferase (GLuc) expression were made (Figure 4a). The promoter cassette with GLuc was then introduced into *Chlamydomonas*. Positive transformants harboring GLuc gene were analyzed by PCR. The expression of GLuc in transgenic *Chlamydomonas* was validated by quantitative real-time PCR (Figure S1). After the expression of GLuc was verified, we compared the strength of *CrepsaD* promoter, which is one of the strongest promoters in *Chlamydomonas*, with *CvpsaD* promoter through luciferase activity assay using 20 randomly selected positive transformants. The luciferase activity operated by *CvpsaD* promoter was 17.3 times lower than that by *CrepsaD* promoter. At the same condition, the non-transgenic control showed negligible luciferase expression (Figure 4b). The intensity of the *CvpsaD* promoter identified through luciferase activity assay was similar to that observed at RNA and protein expression levels, and it was observed to have a lower transformation efficiency as well (Figure S2). In general, heterologous promoters exhibit lower expression efficiency than endogenous promoters. The *CvpsaD* promoter is heterologous to *Chlamydomonas*, and therefore the necessary regulatory elements of the *CvpsaD* promoter may be lacking in *Chlamydomonas* [17]. Moreover, the sequence of *CrepsaD* promoter (Figure S3) differs from that of *CvpsaD* promoter in that there is a TATA box core element near the TSS. Additionally, the CAAT motif, a cis-acting element, is located at about 160 bp upstream of TATA box and 135 bp upstream of TSS. These features observed in the *CrepsaD* promoter sequence are similar to what is already known. Therefore, in *Chlamydomonas*, the presence of a core element neighboring the cis-acting elements on the promoter seems to be an important factor in regulating promoter strength.

### 2.4. Confirmation of the CvpsaD Promoter Functionality by Expression of GFP-GUS Activity in Planta

**Figure 5:**
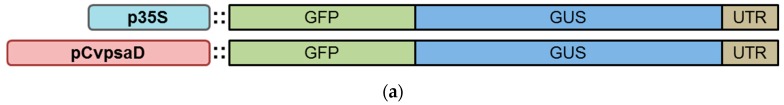

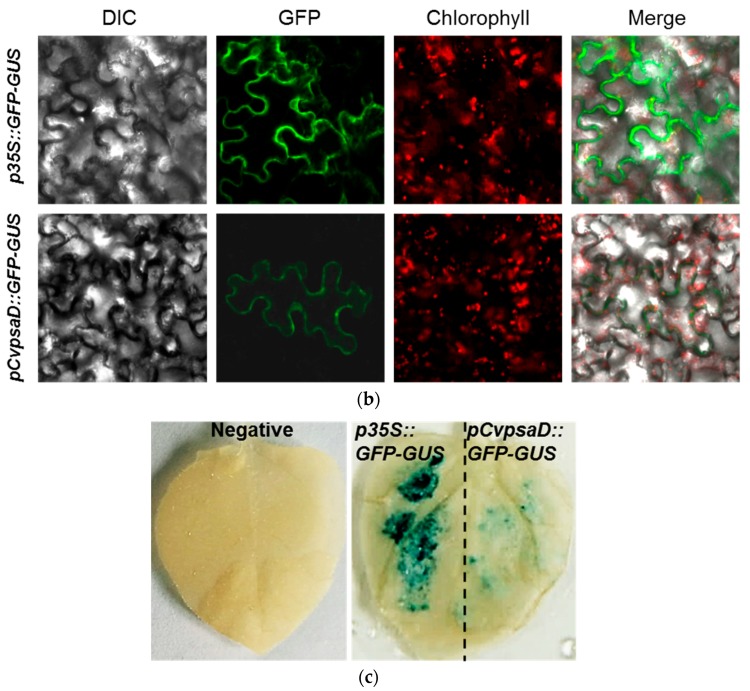
Evaluation of the *CvpsaD* promoter function in higher plants. Green fluorescent protein (GFP) analysis and the β-glucuronidase (GUS) histochemical assay were performed on the infiltrated leaves. (**a**) Schematic representation of promoter cassettes conjugated with the GFP-GUS fusion gene. (**b**) Green fluorescence in epidermal cells and (**c**) GUS reporter assay in leaf tissue of *N. benthamiana*. Transient expression of GFP-GUS three days after infiltration with *Agrobacterium* harboring GFP-GUS vector.

In this experiment, we re-confirmed the function of *CvpsaD* promoter derived from *C. vulgaris* by expression in higher plant. We expected the promoter to be able to express the gene in plants, as the *CvpsaD* sequence is similar to that in *Noccaea caerulescens* (JAU65035.1) (Figure 1). To evaluate the expression of a gene (GFP-fused GUS reporter system) by the *CvpsaD* promoter in a higher plant, transient expression system in *Nicotiana benthamiana* was used. To prepare the GFP-GUS expression cassette, we used pHZM43 vector derived from pGreen. The *CvpsaD* promoter sequence was cloned into 5′ upstream region of the CDS of GFP-GUS (Figure 5a). The cauliflower mosaic virus (CaMV) 35S promoter cassette was used as a positive control. After vector construction, *Agrobacterium* harboring the promoter cassette was cultivated for infiltration, *Agrobacterium* infiltrated *N. benthamiana* leaves were left for 3 days to allow protein expression. Following that, GFP expression (green) and chlorophyll autofluorescence (red) were detected by fluorescence microscopy and GUS activity was analyzed in the same transgenic leaves (Figure 5b,c). After infiltration, green fluorescence and blue color expressed by *pCvpsaD*::*GFP*-*GUS* were detected in epidermal cell cytosol and leaf tissues (mesophyll cell), although the signal was weaker than that of the positive control. This reflects that the strength of the *CvpsaD* promoter is not enough high in *N. benthamiana*. This result is that same as that observed earlier in *C. reinhardtii* using the *pCvpsaD::GLuc* cassette (Figure 4). Computational analysis showed that the CaMV 35S promoter has a core element, such as the TATA box, and a cis-acting element, CAAT motif, distributed around the core element (Figure S3). The characteristics of this sequence are similar to those of *CrepsaD*, as mentioned above. Therefore, this suggests that the absence of a core element in the *CvpsaD* promoter sequence may be the reason for its weak strength.

### 2.5. Light Inducibility of the CvpsaD Promoter under Various Light Conditions

**Figure 6:**
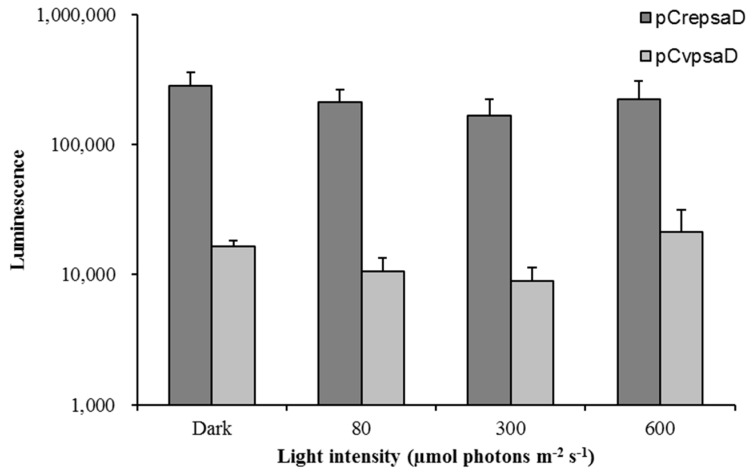
Analysis of the inducibility of the *CvpsaD* promoter under various light conditions. Two different transformants, each harboring one of the promoter cassettes, were used for examination of light response. Luciferase activities were analyzed to determine the light response.

Several light-responsive elements are present on *CvpsaD* promoter, such as the CATT box, G-box, GATA box, MNF1 motif, SORLIP1AT, SORLIP2AT, and the Sp1 motif (Figure 2). Therefore, in order to determine the light responsiveness of the *CvpsaD* promoter, we measured luciferase activity under various light conditions (dark to 600 µmol photons m^−2^ s^−1^) in *C. reinhardtii*. The *CrepsaD* promoter, known as a constitutive promoter [26], was used again as a control in this experiment. In Figure 6, the result shows the difference in luciferase activity affected by the light intensity. Luciferase activity was not significantly increased by high light intensity (300 and 600 µmol photons m^−2^ s^−1^). This result suggests that despite the presence of light-responsive elements on the *CvpsaD* promoter, it is not affected by light. According to a previous report [29,49], the SORLIP motif (GCCAC or GGGCC), known as the overrepresented element near TSS in light-induced genes, plays an important role in determining the reactivity to light. Also, all the other light-responsive elements described above on the *CvpsaD* promoter are also known to be involved in the light responsiveness of the genes in plants. However, in our experiment, the SORLIP motif, as well as all other light-responsive elements seemed to be ineffective for induction of a light response in *C. reinhardtii*. In the previous study, it has been reported that *psaD* gene of spinach is expressed in response to light, but the *psaD* promoter was insensitive to light [30]. This difference between a gene and promoter response to light can be interpreted as being due to a particular sequence present in the coding region or further downstream [30]. This result shows that a computational analysis using already known motifs cannot be an absolute factor in understanding the function of the new promoter, thus independent and extensive experimental results for each promoter should be carried out.

## 3. Materials and Methods

### 3.1. Microalgae Cell Culture

*Chlorella vulgaris* UTEX395 (purchased from UTEX, Culture Collection of Algae, Austin, TX, USA) and *Chlamydomonas reinhardtii* CC-4349 cw15 mt- (kindly provided by Dr. Jae-Hyeok Lee, University of British Columbia) were maintained photoheterotrophically in Tris-acetate-phosphate (TAP) medium at 25 °C and continuous light (50–70 μmol photons m^−2^ s^−1^) conditions on an orbital shaker with shaking at 90 rpm. For selection and maintenance of transgenic cells, TAP medium was fortified with paromomycin (25 µg/mL) or hygromycin-B (25 µg/mL).

### 3.2. Isolation and Sequence Analysis of Putative CvpsaD Gene

Putative *psaD* sequence from *Chlorella vulgaris* was predicted by a BLAST search (TBLASTN), based on the *CrepsaD* amino acid sequence (Accession: AAL73208.1). A homology analysis of amino acid sequence was conducted using NEW GENESCAN Web server (http://genes.mit.edu/GENSCAN.html) to determine the CDS of *CvpsaD*. The 570 bp full length complementary DNA (cDNA) of the putative *CvpsaD* gene was amplified and sequenced using specific primers (Table S1). The alignment of *psaD* amino acid sequence from various species was performed by NCBI BLAST (https://blast.ncbi.nlm.nih.gov/Blast.cgi?CMD=Web&PAGE_TYPE=BlastHome) and illustrated using a multiple sequence alignment tool (http://www.jalview.org/). Moreover, 5′ and 3′ RACE was performed for identification of whole mRNA transcript using the 5′/3′ RACE kit (2nd Generation kit, Roche, Germany), according to the manufacturer’s protocol. The location and distribution of putative cis-acting elements in *CvpsaD* promoter was analyzed using PLACE (https://sogo.dna.affrc.go.jp/cgi-bin/sogo.cgi?lang=en&pj=640&action=page&page=newplace), PlantCARE (http://bioinformatics.psb.ugent.be/webtools/plantcare/html), and Promoter 2.0 Prediction Server (http://www.cbs.dtu.dk/services/Promoter/).

### 3.3. Expression Vector Construction

The *CvpsaD* promoter was conjugated with *aphVIII* or GLuc vector using restriction enzyme and T4 DNA ligase, according to the protocols of the traditional cloning method. For functionality analysis of the *CvpsaD* promoter, the *aphVIII* gene was cloned into pChlamy3 plus vector, which is derived from pChamy3 vector after deleting the AR promoter [29]. The promoter sequence was then sub-cloned upstream of *aphVIII*::3′ UTR at the *Spe*I and *Kpn*I restriction sites. The p*CrepsaD*::*aphVIII* cassette was created following the same procedure (Figure 3). For analysis of the strength of the *CvpsaD* promoter, the GLuc cassette was developed by replacing *aphVIII* with GLuc. *aphVIII* was removed from the promoter::aphVIII cassette by double digestion with *Kpn*I and *Not*I, and GLuc was then cloned into the cleaved region by T4 DNA ligase. For *Nicotiana* transformation, the pHZM43 vector derived from pGreen vector was used. The *CvpsaD* promoter sequence was cloned upstream of GFP-GUS fusion gene at the *Kpn*I and *Xho*I restriction sites. All primer sequences are listed in Table S1.

### 3.4. Generation of Transgenic Microalgae

To develop transgenic *C. reinhardtii*, transformation was performed by the glass bead method using the promoter cassette DNA, as previously described [29,50]. *C. reinhardtii* cells were grown up to mid-log phase in TAP (Tris-Phosphate-Phosphate) medium, and transformation was performed with 1 μg linearized DNA. After transformation, whole cells were spread on TAP agar plates containing antibiotics (25 μg/mL paromomycin or 25 µg/mL hygromycin-B). Antibiotic resistant colonies were selected within 10 days. The transformants were confirmed by colony PCR [41]. Transformation efficiency was analyzed by electroporation. Electroporation was performed using the Bio-Rad Gene Pulser X cell apparatus (Bio-Rad, Hercules, CA, USA) with 40 mM sucrose, following the protocol of GeneArt^®^ *Chlamydomonas* Engineering Kits (Life Technologies, Camarillo, CA, USA).

### 3.5. Functional Analysis of the CvpsaD Promoter in Microalgae

#### 3.5.1. Quantitative Real-Time PCR for Analysis of Transgene RNA Expression

The RNA expression of *aphVIII* or GLuc was determined using quantitative real-time PCR (qPCR) performed by TaKaRa PCR Thermal Cycler Dice (Takara, Shiga, Japan). *aphVIII* or GLuc were amplified with specific primers (Table S1), and *RACK1* gene served as the internal control [51]. Raw data of qPCR was calculated by ΔΔ*C*t method using the software provided by the manufacturer. RNA expression level of gene was normalized to that of *RACK1*. RNA expression levels for the *CrepsaD* and *CvpsaD* promoters were compared.

#### 3.5.2. Western Blot Analysis

Total cells (2.5 × 10^6^ cells) were boiled in sodium dodecyl sulfate-polyacrylamide gel electrophoresis (SDS-PAGE) loading buffer and separated on SDS-polyacrylamide gels (12%), and then electro blotted onto a polyvinylidene fluoride (PVDF) membrane using the Semi-Dry Transfer Cell (Bio-Rad, Berkeley, CA, USA). GLuc protein was detected using primary rabbit anti-GLuc antibody (NEB, Beverly, MA, USA) and secondary HRP-conjugated goat anti-rabbit IgG (H + L) antibody (Life Technologies, Carlsbad, CA, USA). GLuc protein was visualized on X-ray film by chemiluminescence using the EPD western reagent (ELPIS-BIOTECH, Daejeon, Korea).

#### 3.5.3. Luciferase Assay

To measure the luciferase activity, the cell density of each transgenic cell culture was so adjusted that optical density (OD)_750nm_ = 0.5 at the mid-log phase. Cells were harvested from 1 mL of cell culture by centrifugation (4000× *g* for 3 min) and used for the luciferase activity assay. Luciferase activity was measured using the *Renilla* Luciferase Assay Kit (Promega, Madison, WI, USA), according to manufacturer’s protocol with slight modifications [41]. Cell pellets were resuspended in 100 μL of cell lysis buffer and mixed vigorously by vortexing for 2–3 min. The resuspension was then centrifuged at 13,000 rpm for 5 min at 4 °C. After centrifugation, 90 µL of the supernatant was transferred to a new tube and 10 µL of luciferase substrate was added to it. After mixing the supernatant and substrate, the luminescence was measured immediately using Glo Max™ 20/20 (Promega, Fitchburg, WI, USA). The luciferase assay was repeated three times.

### 3.6. Analysis of the Function of the CvpsaD Promoter in Plants

*N. benthamiana* leaves were infiltrated with *A. tumefaciens* strain GV3101:pMP90 harboring *p35S*::*GFP-GUS* or *pCvpsaD*::*GFP-GUS*. Transiently expressed proteins were analyzed by fluorescence microscopy and the GUS reporter assay. Green fluorescence and chlorophyll autofluorescence were detected in the infiltrated leaves using Nikon Eclipse Ni-E microscopy with DS-Qi2 camera (Nikon, Tokyo, Japan), 2.5–3 days after infiltration. Fluorescence detection wavelength ranged from 535 ± 50 nm with FITC filter for GFP and from 700 ± 75 nm with Cy5 filter for the chloroplast autofluorescence. The infiltrated leaves were also histochemically assayed for GUS activity [52]. Post-acquisition image processing involved contrast change only.

### 3.7. Analysis of Light Inducibility of the CvpsaD Promoter

For light inducibility analysis, transgenic cells harboring the *pCvpsaD*::*GLuc* cassette were placed under various constant light conditions (dark, 80, 300, and 600 μmol photons m^−2^ s^−1^) for 2 h [29]. After the exposure to different light intensities, the cell density was adjusted to equal OD value (OD_750nm_ = 0.5) and cells were then spun down by centrifugation (4000× *g* for 3 min). Luciferase activity was measured, according to the procedure described above using the *Renilla* Luciferase Assay Kit (Promega, Fitchburg, WI, USA). Light inducibility analysis was performed with two different transgenic lines.

## 4. Conclusions

The development of a suitable promoter is one of the basic essentials in the molecular toolbox for genetic modification. In this study, the promoter of photosystem I protein D (*psaD*) gene was identified from the whole genome sequence of *Chlorella vulgaris* UTEX395 by computational analysis. And, the function of the new promoter derived from *C. vulgaris* was verified using model algal strain and higher plant. While its verification directly in *Chlorella* is still a major challenge, we suggest that the *CvpsaD* promoter can be used as an effective tool for genetic modification of *Chlorella* species. Moreover, it should be noted that this is the first report on a promoter derived from *C. vulgaris* that can be used to drive gene expression in green microalgae, as well as in higher plants. Therefore, *CvpsaD* promoter investigated in this study could be an efficient tool for the transformation of *C. vulgaris* through the further optimization of this *CvpsaD* promoter by several engineering strategies such as the determination of the most effective size of the promoter, a combination with an appropriate terminator or development of fusion promoter.

## Figures and Tables

**Table 1:** List of putative cis-acting elements in the *psaD* promoter in *Chlorella vulgaris*.

No	Name	Sequence	Function
1	BRE	GGGCGCC	TFIIB recognition element
2	CAAT box	GGCAAT CAAT CAAAT	Common cis-acting element in promoter and enhancer regions
3	CCAAT box	CAACGG	MYBHv1 binding site
4	CATT box	GCATTC	Part of light-responsive element
5	CCCAT motif	CCCAT	Putative core element, sap
6	CGTCA-motif	CGTCA	MeJA-responsiveness
7	DPE	GGCACC	Downstream promoter element, requires an Inr
8	G-box	CACGTG	cis-acting regulatory element involved in light responsiveness
9	GATA box	GATA	Binding with ASF-2, required for high level, light-regulated, and tissue-specific expression
10	GT-1 motif	GATAAT GAAAA	Pathogen- and salt-induced element
11	Inr	CTCATTCC	Initiator element, core promoter element
12	MNF1	GTGCCCTAAAGCTGCA	Light-responsive element
13	SORLIP1AT/2AT	GCCAC GGGCC	Light-responsive elements
14	Sp1	CCACCC	Light-responsive element
15	TATA box	TATA	Core promoter element around −30 of transcription start
16	TGACG-motif	TGACG	MeJA-responsiveness
17	W box	TGACT TTGAC	cis-regulatory element, recognized by WRKY transcription factors, stress responsive
18	5’ untranslated region (UTR) Py-rich stretch	TTTCTTCTGT	Confers high transcription levels without the need for other upstream cis-acting elements

## Supplementary Materials

The following are available online at http://www.mdpi.com/1422-0067/19/7/1969/s1.

## References

[1] Pulz O., Gross W. (2004). Valuable products from biotechnology of microalgae. Appl. Microbiol. Biotechnol..

[2] Stengel D.B., Connan S., Popper Z.A. (2011). Algal chemodiversity and bioactivity: Sources of natural variability and implications for commercial application. Biotechnol. Adv..

[3] Leu S., Boussiba S. (2014). Advances in the production of high-value products by microalgae. Ind. Biotechnol..

[4] Yu X., Chen L., Zhang W. (2015). Chemicals to enhance microalgal growth and accumulation of high-value bioproducts. Front. Microbiol..

[5] Gimpel J.A., Henríquez V., Mayfield S.P. (2015). In metabolic engineering of eukaryotic microalgae: Potential and challenges come with great diversity. Front. Microbiol..

[6] Liang Y., Sarkany N., Cui Y. (2009). Biomass and lipid productivities of *Chlorella vulgaris* under autotrophic, heterotrophic and mixotrophic growth conditions. Biotechnol. Lett..

[7] Perez-Garcia O., Escalante F.M., de-Bashan L.E., Bashan Y. (2011). Heterotrophic cultures of microalgae: Metabolism and potential products. Water Res..

[8] Yang B., Liu J., Jiang Y., Chen F. (2016). Chlorella species as hosts for genetic engineering and expression of heterologous proteins: Progress, challenge and perspective. Biotechnol. J..

[9] Chow K.-C., Tung W. (1999). Electrotransformation of *Chlorella vulgaris*. Plant Cell Rep..

[10] Kim D.-H., Kim Y.T., Cho J.J., Bae J.-H., Hur S.-B., Hwang I., Choi T.-J. (2002). Stable integration and functional expression of flounder growth hormone gene in transformed microalga, chlorella ellipsoidea. Mar. Biotechnol..

[11] Liu L., Wang Y., Zhang Y., Chen X., Zhang P., Ma S. (2013). Development of a new method for genetic transformation of the green alga chlorella ellipsoidea. Mol. Biotechnol..

[12] Ng S.L., Harikrishna J.A., Bakar F.A., Yeo C.C., San Cha T. (2016). Heterologous expression of the streptococcus pneumoniae yoeb and pezt toxin genes is lethal in *Chlorella vulgaris*. Algal Res..

[13] San Cha T., Yee W., Aziz A. (2012). Assessment of factors affecting agrobacterium-mediated genetic transformation of the unicellular green alga, *Chlorella vulgaris*. World J. Microbiol. Biotechnol..

[14] Liu J., Sun Z., Gerken H., Huang J., Jiang Y., Chen F. (2014). Genetic engineering of the green alga chlorella zofingiensis: A modified norflurazon-resistant phytoene desaturase gene as a dominant selectable marker. Appl. Microbiol. Biotechnol..

[15] Run C., Fang L., Fan J., Fan C., Luo Y., Hu Z., Li Y. (2016). Stable nuclear transformation of the industrial alga chlorella pyrenoidosa. Algal Res..

[16] Talebi A.F., Tohidfar M., Tabatabaei M., Bagheri A., Mohsenpor M., Mohtashami S.K. (2013). Genetic manipulation, a feasible tool to enhance unique characteristic of *Chlorella vulgaris* as a feedstock for biodiesel production. Mol. Biol. Rep..

[17] Thanh T., Chi V.T.Q., Omar H., Abdullah M.P., Napis S. (2012). Sequence analysis and potentials of the native rbcs promoter in the development of an alternative eukaryotic expression system using green microalga ankistrodesmus convolutus. Int. J. Mol. Sci..

[18] Seo S., Jeon H., Hwang S., Jin E., Chang K.S. (2015). Development of a new constitutive expression system for the transformation of the diatom phaeodactylum tricornutum. Algal Res..

[19] Hallmann A. (2007). Algal transgenics and biotechnology. Transgen. Plant J..

[20] Franklin S., Ngo B., Efuet E., Mayfield S.P. (2002). Development of a gfp reporter gene for chlamydomonas reinhardtii chloroplast. Plant J..

[21] Radakovits R., Jinkerson R.E., Darzins A., Posewitz M.C. (2010). Genetic engineering of algae for enhanced biofuel production. Eukaryot. Cell.

[22] Kucho K.-I., Kakoi K., Yamaura M., Iwashita M., Abe M., Uchiumi T. (2013). Codon-optimized antibiotic resistance gene improves efficiency of transient transformation in frankia. J. Biosci..

[23] Shao N., Bock R. (2008). A codon-optimized luciferase from gaussia princeps facilitates the in vivo monitoring of gene expression in the model alga chlamydomonas reinhardtii. Curr. Genet..

[24] Lumbreras V., Stevens D.R., Purton S. (1998). Efficient foreign gene expression in chlamydomonas reinhardtii mediated by an endogenous intron. Plant J..

[25] Schroda M., Blöcker D., Beck C.F. (2000). The hsp70a promoter as a tool for the improved expression of transgenes in chlamydomonas. Plant J..

[26] Kumar A., Falcao V.R., Sayre R.T. (2013). Evaluating nuclear transgene expression systems in chlamydomonas reinhardtii. Algal Res..

[27] Wu J., Hu Z., Wang C., Li S., Lei A. (2008). Efficient expression of green fluorescent protein (gfp) mediated by a chimeric promoter in chlamydomonas reinhardtii. Chin. J. Oceanol. Limnol..

[28] Scranton M.A., Ostrand J.T., Georgianna D.R., Lofgren S.M., Li D., Ellis R.C., Carruthers D.N., Dräger A., Masica D.L., Mayfield S.P. (2016). Synthetic promoters capable of driving robust nuclear gene expression in the green alga chlamydomonas reinhardtii. Algal Res..

[29] Baek K., Lee Y., Nam O., Park S., Sim S.J., Jin E. (2016). Introducing dunaliella lip promoter containing light-inducible motifs improves transgenic expression in chlamydomonas reinhardtii. Biotechnol. J..

[30] Flieger K., Wicke A., Herrmann R., Oelmüller R. (1994). Promoter and leader sequences of the spinach psad and psaf genes direct an opposite light response in tobacco cotyledons: Psad sequences downstream of the atg codon are required for a positive light response. Plant J..

[31] Chitnis V.P., Ke A., Chitnis P.R. (1997). The psad subunit of photosystem i (mutations in the basic domain reduce the level of psad in the membranes). Plant Physiol..

[32] Fischer N., Rochaix J.-D. (2001). The flanking regions of psad drive efficient gene expression in the nucleus of the green alga chlamydomonas reinhardtii. Mol. Genet. Genom..

[33] Nakamura M., Tsunoda T., Obokata J. (2002). Photosynthesis nuclear genes generally lack tata-boxes: A tobacco photosystem i gene responds to light through an initiator. Plant J..

[34] Xi H., Yu Y., Fu Y., Foley J., Halees A., Weng Z. (2007). Analysis of overrepresented motifs in human core promoters reveals dual regulatory roles of yy1. Genome Res..

[35] Butler J.E., Kadonaga J.T. (2002). The rna polymerase ii core promoter: A key component in the regulation of gene expression. Genes Dev..

[36] Zhang J., Zhang X., Wang Y., Hou H., Qian Y. (2012). Characterization of sequence elements from malvastrum yellow vein betasatellite regulating promoter activity and dna replication. Virol. J..

[37] Ganguli S., Das S.G., Chakraborty H.J., Gupta S., Datta A. (2013). Identification of regulatory sequence signatures in microrna precursors implicated in neurological disorders. Adv. Biosci. Biotechnol..

[38] Li T., Gong C., Wang T. (2010). The rice light-regulated gene ra68 encodes a novel protein interacting with oxygen-evolving complex psbo mature protein. Plant Mol. Biol. Rep..

[39] Teakle G.R., Manfield I.W., Graham J.F., Gilmartin P.M. (2002). Arabidopsis thaliana gata factors: Organisation, expression and dna-binding characteristics. Plant Mol. Biol..

[40] Hudson M.E., Quail P.H. (2003). Identification of promoter motifs involved in the network of phytochrome a-regulated gene expression by combined analysis of genomic sequence and microarray data. Plant Physiol..

[41] Park S., Lee Y., Lee J.H., Jin E. (2013). Expression of the high light-inducible dunaliella lip promoter in chlamydomonas reinhardtii. Planta.

[42] Ahrazem O., Rubio-Moraga A., López R.C., Gómez-Gómez L. (2009). The expression of a chromoplast-specific lycopene beta cyclase gene is involved in the high production of saffron’s apocarotenoid precursors. J. Exp. Bot..

[43] Li M., Cui Y., Gan Z., Shi C., Shi X. (2015). Isolation and analysis of the cppsy gene and promoter from chlorella protothecoides cs-41. Mar. Drugs.

[44] Park H.C., Kim M.L., Kang Y.H., Jeon J.M., Yoo J.H., Kim M.C., Park C.Y., Jeong J.C., Moon B.C., Lee J.H. (2004). Pathogen-and nacl-induced expression of the scam-4 promoter is mediated in part by a gt-1 box that interacts with a gt-1-like transcription factor. Plant Physiol..

[45] Rushton P.J., Somssich I.E., Ringler P., Shen Q.J. (2010). Wrky transcription factors. Trends Plant Sci..

[46] Liu J., Wang F., Yu G., Zhang X., Jia C., Qin J., Pan H. (2015). Functional analysis of the maize c-repeat/dre motif-binding transcription factor cbf3 promoter in response to abiotic stress. Int. J. Mol. Sci..

[47] Niu G.-L., Gou W., Han X.-L., Qin C., Zhang L.-X., Abomohra A.E.-F., Ashraf M. (2018). Cloning and functional analysis of phosphoethanolamine methyltransferase promoter from maize (*Zea mays* L.). Int. J. Mol. Sci..

[48] Berthold P., Schmitt R., Mages W. (2002). An engineered streptomyces hygroscopicus aph 7 gene mediates dominant resistance against hygromycin b in chlamydomonas reinhardtii. Protist.

[49] Jiao Y., Ma L., Strickland E., Deng X.W. (2005). Conservation and divergence of light-regulated genome expression patterns during seedling development in rice and arabidopsis. Plant Cell.

[50] Kindle K.L. (1990). High-frequency nuclear transformation of chlamydomonas reinhardtii. Proc. Natl. Acad. Sci. USA.

[51] Shtaida N., Khozin-Goldberg I., Solovchenko A., Chekanov K., Didi-Cohen S., Leu S., Cohen Z., Boussiba S. (2014). Downregulation of a putative plastid pdc e1α subunit impairs photosynthetic activity and triacylglycerol accumulation in nitrogen-starved photoautotrophic chlamydomonas reinhardtii. J. Exp. Bot..

[52] Jefferson R.A. (1987). Assaying chimeric genes in plants: The gus gene fusion system. Plant Mol. Biol. Rep..

