# Supplementary material for: Identification and Functional Analysis of the psaD Promoter of Chlorella vulgaris Using Heterologous Model Strains
Source: Int J Mol Sci. 2018 Jul 6;19(7):1969. doi: 10.3390/ijms19071969 (PMC6073903; doi:10.3390/ijms19071969)
Supplement: Supplementary file 1 [file ijms-19-01969-s001.pdf]

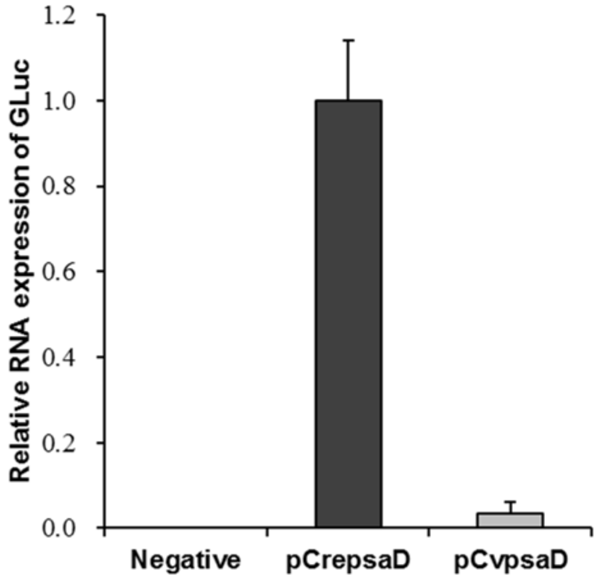

Figure S1. Verification of the expression of Gaussia luciferase (GLuc) gene by promoters. Relative RNA expression level of GLuc by *CvpsaD* promoters compared to *CrepsaD* promoter.

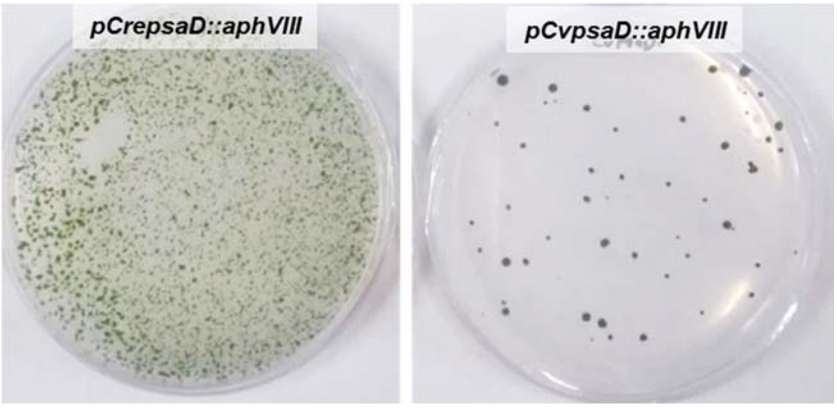

Figure S2. Transformation efficiency of the promoter cassettes, *pCrepsaD::aphVIII* and *pCvpsaD::aphVIII*. Different numbers of colonies were present on selective agar plate against paromomycin.

(a)

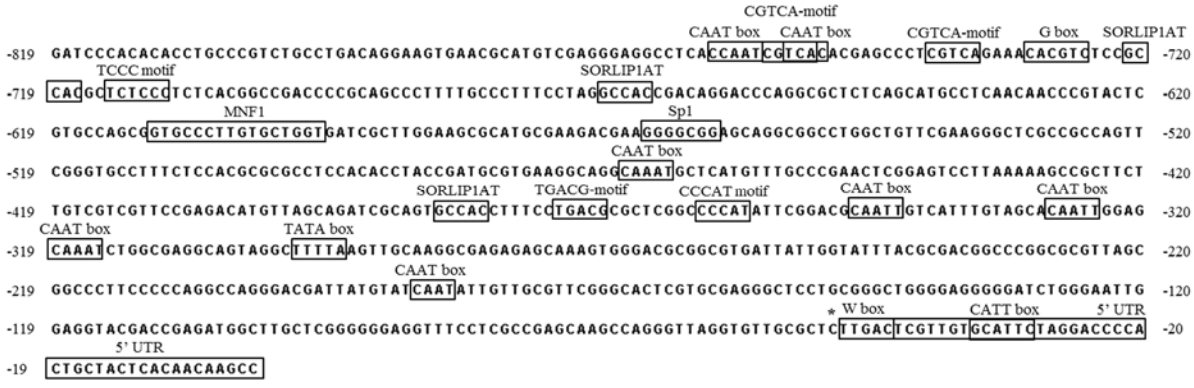

(b)

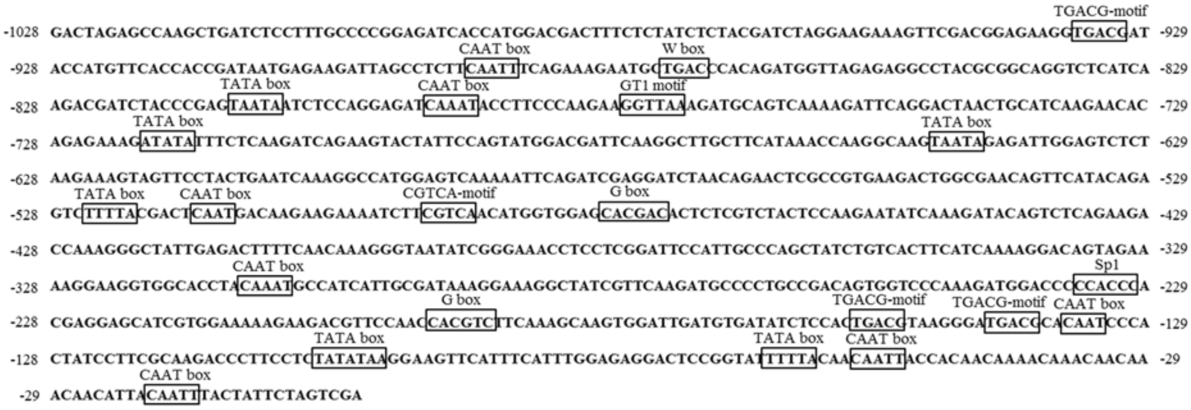

Figure S3. Nucleotide sequence of the (a) *CrepsaD* promoter and (b) CaMV 35S promoter. The putative transcriptional start site (TSS) is indicated by an asterisk. All putative *cis*-acting elements are boxed and labeled.

Table S1. List of primer sequences used in this study.

Name	Sequence (5′ → 3′)	Specification
pCvpsaD-clone-F	GGACTAGTGTCACAGGCCGTCTCGCAGT	Clone *CvpsaD* promoter into expression vector for *Chlamydomonas*.
pCvpsaD-clone-R	CGGGGTACCGGTGCCAAGGACAAGCTGGATG	
pCrepsaD-clone-F	GGACTAGTGATCCACACACCTGCCGTC	Clone *CrepsaD* promoter into expression vector for *Chlamydomonas*.
pCrepsaD-clone-R	CGGGGTACCGGCTTGTTGTGAGTAGCAGTG	
aph VIII-insert-F	CCTCGGGGGCTGGTGTTTATCG	Clone *aphVIII* gene into expression vector for *Chlamydomonas*.
aph VIII-insert-R	CACCCCTTCCGCTCCTCGTCCAGA	
RACK1-F	GGCTGGGACAAGATGGTCAA	Reference gene of mRNA expression in *Chlamydmonas*
RACK1-R	GAGAAGCACAGGCAGTGGAT	
aph VIII-RT-F	AGGATCTGGCGGTTTGCCAC	Confirm RNA level of *aphVIII*.
aph VIII-RT-R	GAACCACGGGTCCTCCTCGT	
GLUC-insert-F	GGGGTACCATGGTCAACGGCGTGAAGGTGCTG	Clone GLuc gene into expression vector for *Chlamydomonas*.
GLUC-insert-R	GGAATTCCATATGTTACGTATCGTCGCCGCCGGC	
GLUC-qPCR-F	GGCGAGGCCATCGTGGACAT	Measure mRNA level of GLuc.
GLUC-qPCR-R	TGGCCCTGGATCTTGCTGGC	
pCvpsaD-clone2-F	GGGGTACCGTCACAGGCCGTCTCGCAG	Clone *CvpsaD* promoter into expression vector for tobacco.
pCvpsaD-clone2-R	CCCGCTCGAGGGTGCCAAGGACAAGCTGGATG	
